# Transcriptome Profiling of Prostate Cancer, Considering Risk Groups and the TMPRSS2-ERG Molecular Subtype

**DOI:** 10.3390/ijms24119282

**Published:** 2023-05-25

**Authors:** Anastasiya A. Kobelyatskaya, Elena A. Pudova, Irina V. Katunina, Anastasiya V. Snezhkina, Maria S. Fedorova, Vladislav S. Pavlov, Anastasiya O. Kotelnikova, Kirill M. Nyushko, Boris Y. Alekseev, George S. Krasnov, Anna V. Kudryavtseva

**Affiliations:** 1 Engelhardt Institute of Molecular Biology, Russian Academy of Sciences, 119991 Moscow, Russia; pudova_elena@inbox.ru (E.A.P.);; 2 National Medical Research Radiological Center, Ministry of Health of the Russian Federation, 125284 Moscow, Russia

**Keywords:** prostate cancer, RNA-Seq, transcriptome, TMPRSS2-ERG, heterogeneity, risk groups, molecular subtype, ISUP, gene expression, pathways

## Abstract

Molecular heterogeneity in prostate cancer (PCa) is one of the key reasons underlying the differing likelihoods of recurrence after surgical treatment in individual patients of the same clinical category. In this study, we performed RNA-Seq profiling of 58 localized PCa and 43 locally advanced PCa tissue samples obtained as a result of radical prostatectomy on a cohort of Russian patients. Based on bioinformatics analysis, we examined features of the transcriptome profiles within the high-risk group, including within the most commonly represented molecular subtype, TMPRSS2-ERG. The most significantly affected biological processes in the samples were also identified, so that they may be further studied in the search for new potential therapeutic targets for the categories of PCa under consideration. The highest predictive potential was found with the *EEF1A1P5*, *RPLP0P6*, *ZNF483*, *CIBAR1*, *HECTD2*, *OGN*, and *CLIC4* genes. We also reviewed the main transcriptome changes in the groups at intermediate risk of PCa—Gleason Score 7 (groups 2 and 3 according to the ISUP classification)—on the basis of which the *LPL*, *MYC*, and *TWIST1* genes were identified as promising additional prognostic markers, the statistical significance of which was confirmed using qPCR validation.

## 1. Introduction

Prostate cancer (PCa) is one of the most common types of cancer among men worldwide [1]. The majority of PCa cases are diagnosed as having a localized form, which represents the early malignant process confined to the prostate gland without spreading beyond its borders. In the early stages of localized PCa (LPCa), symptoms may include minor changes in the urinary system or even no symptoms at all. The standard clinical diagnosis of LPCa includes various methods such as prostate palpation, measurement of levels of prostate-specific antigen (PSA), ultrasound/magnetic resonance imaging, and biopsy.

LPCa often has a favorable prognosis, and there is a wide range of available therapeutic approaches (active surveillance, radiation therapy, radical prostatectomy, or their combinations) that can be chosen based on the patient’s risk stratification for biochemical recurrence. One of the main systems for classifying risk groups in PCa is the D’Amico classification system, which defines three risk groups: low, intermediate, and high. This risk group assessment is based on three factors: the Gleason score (GS), PSA level, and stage of the disease. The low-risk group is characterized by a GS ≤ 6, PSA level < 10 ng/mL, and T1–T2a stage; intermediate risk by GS = 7, PSA level 10–20 ng/mL, and/or T2b–T2c stage; and high risk by GS ≥ 8, PSA level > 20 ng/mL, and/or stage ≥ T3a [2].

Patient stratification into risk groups is a widely used approach for assessing the prognosis of PCa. However, in some cases, this method may not be sufficiently informative, potentially leading to incorrect conclusions and inappropriate treatments. One of the limitations of patient stratification into such risk groups is that this method does not consider many other factors that may affect the disease prognosis, such as age, comorbidities, and genetics. Studies have shown that even patients with low D’Amico risk may have a poor prognosis if they have other risk factors that were not considered during stratification [3].

Thus, stratification of patients into risk groups is a useful tool for assessing PCa prognosis, but other risk factors and individual patient characteristics must also be considered. 

One of the main factors complicating the diagnosis and treatment of PCa, as well as of other types of cancer, is molecular heterogeneity. This phenomenon is defined by differences in the molecular properties of tumor cells, such as changes in gene expression, mutations, and other factors that affect cell function and behavior [4,5]. Furthermore, the development of aggressive forms of PCa requires only a few driver alterations [6,7,8]. Molecular heterogeneity is also present within LPCa, and this can lead to different prognosis and outcomes [4,5].

It has been shown that 74% of all PCa cases can be attributed to one of a range of molecular subtypes that have been identified on the basis of analysis of somatic mutations, changes in copy number, gene expression, gene fusions, and DNA methylation [9].

There are currently seven major molecular subtypes of PCa that have been identified as part of the Prostate Adenocarcinoma Project of The Cancer Genome Atlas (TCGA) consortium [9]. Four of the seven subtypes are characterized by the presence of fusion transcripts between the *TMPRSS2* gene exons and the exons of the *ETS* family of genes (the erythroblast transformation-specific family of transcription factors): *ERG*, *ETV1*, *ETV4*, and *FLI1* (the frequency of occurrence of these subtypes is 46%, 8%, 4%, and 1%, respectively). Three other subtypes are characterized by the presence of point mutations in one of the following genes: *SPOP*, *FOXA1*, or *IDH1* (the frequency of occurrence of these subtypes is 11%, 3%, and 1%, respectively) [9,10]. Thus, about half of all cases of prostate cancer have a fused TMPRSS2-ERG transcript, which is formed due to an intrachromosomal rearrangement leading to the fusion of two genes: *TMPRSS2* and *ERG*.

Considering the molecular heterogeneity of PCa in particular, the selection of the group of tumors characterized by the TMPRSS2-ERG fusion transcript allows researchers to focus on specific molecular subtypes of PCa and, therefore, form a more homogeneous group of PCa cases. To improve prognoses and to determine the best treatment approach for each patient with LPCa, a more detailed study of tumor molecular heterogeneity is necessary. New biomarkers and genomic technologies can help in this direction and allow for the identification of the biological nature of any given PCa and its prognosis [11].

This study involved RNA-Seq profiling of 58 LPCa samples from intermediate- and high-risk groups and 43 locally advanced PCa (LAPCa) samples from a cohort of Russian patients, with the aim of identifying transcriptional profile differences between these groups, taking into account the TMPRSS2-ERG subtype, and searching for promising genes as prognostic markers.

## 2. Results

### 2.1. Differentially Expressed Genes among Risk Groups within Localized PCa

LPCa can be classified into one of three risk groups: low, intermediate, and high. Based on the LPCa cases we analyzed, the low-risk group is quite rare (about 2%), whereas the intermediate one is the most frequent (80–82%), and the high-risk group accounts for 16–18%.

Initially, we compared primary PCa tumors belonging to the intermediate- (n = 47) and high-risk (n = 10) groups. Based on RNA-Seq data obtained for PCas in Russian patients having tumors of the high and intermediate LPCa risk groups, just six (↓*COL17A1*, ↑*FAM83D*, ↑*GCSAML*, ↓*IER2*, ↑*IFI44L*, ↓*MYH3*) differentially expressed (DE) genes were identified (*p*-value ≤ 0.05 according to the QLF and MW tests, Log2CPM ≥ 3, |Log2FC| ≥ 1, Supplementary Table S1-1). This is definitely too small a number of DE genes; apparently, these risk groups do not have extensive transcriptomic variations among themselves.

### 2.2. Differentially Expressed Genes between LAPCa and LPCa for High-Risk Group

At this stage of our work, besides the LPCa samples, we included LAPCa cases in the study, in order to find transcriptome differences between these grades of tumor extension.

**Figure 1:**
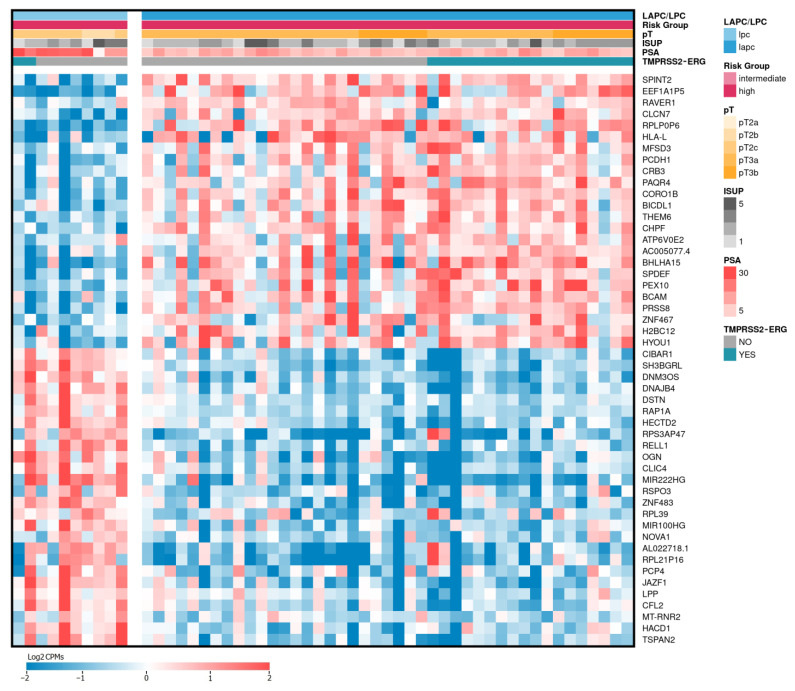
Heatmap of top 50 differentially expressed genes between LAPCa and LPCa. LAPCa—locally advanced PCa; LPCa—localized PCa. Red color indicates upregulated genes, blue—downregulated.

Including the expression data of LPCa cases, as well as the data obtained in our previous work devoted to LAPCa samples, we performed differential expression analysis (DEA) between the groups of LPCa (n = 10) and LAPCa (n = 43) samples classified as high-risk. As a result, 243 DE genes were identified (*p*-values ≤ 0.05 according to the QLF and MW tests, Log2CPM ≥ 3, |Log2FC| ≥ 1, Supplementary Table S1-2). Figure 1 shows the expression profiles of the top 50 DE genes.

**Figure 2:**
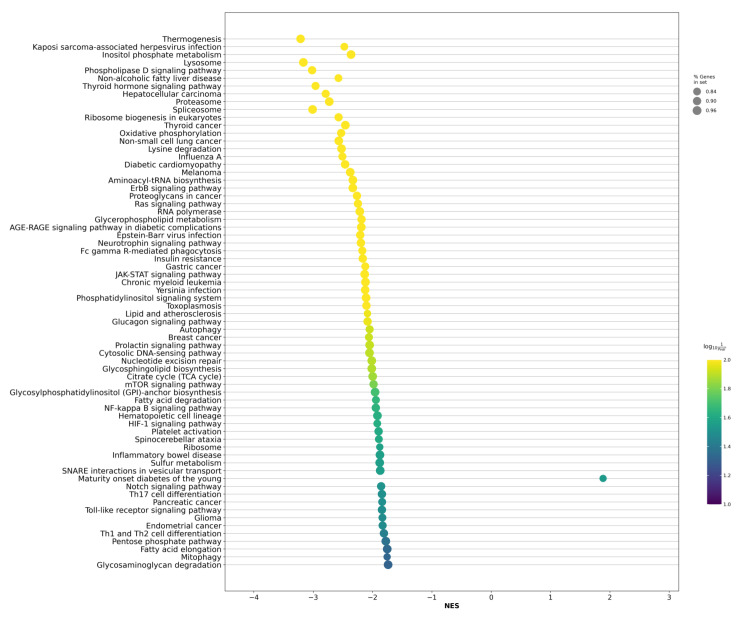
Dotplot of GSEA results illustrating the KEGG-based biological processes associated with the LAPCa category versus LPCa within the high-risk group. The figure shows statistically significant enriched biological pathways based on coexpressed genes. NES—normalized enrichment score.

Based on the identified DE gene profile, we further analyzed the enrichment of biological pathways associated with LAPCa versus LPCa within the high-risk group. As a result of the analysis based on the GSEA algorithm and the KEGG Human 2021 database, we found statistically significant changes in 68 biological processes (FDR ≤ 0.05, Figure 2, Supplementary Table S2-1).

The identified biological pathways include many processes with known involvement in the development and progression of various types of tumors, including “Non-small cell lung cancer”, “Thyroid cancer”, and “Melanoma”, as well as the effects of “Proteoglycans in cancer” and the “Ras signaling pathway”. A complete list of analysis results is presented in Supplementary Table S2-1.

### 2.3. Differentially Expressed Genes between LAPCa and LPCa within the TMPRSS2-ERG Molecular Subtype

It is known that the frequency of occurrence of the TMPRSS2-ERG subtype varies from 40% to 50% in PCa [9,10]. In addition, most researchers consider TMPRSS2-ERG as a factor involved in increased aggressiveness, propensity for invasion, and metastasis [12,13,14]. However, several studies have demonstrated that TMPRSS2-ERG is either a precursor of a good prognosis or has no association with progression and prognosis at all [15,16]. In the present study, the incidence of the TMPRSS2-ERG subtype was about 42% for LAPCa and 28% for LPCa. Furthermore, we searched for associations of the TMPRSS2-ERG subtype with clinical and pathomorphological criteria. Spearman’s correlation analysis did not show a significant association of the TMPRSS2-ERG subtype with any of the following criteria: age, tumor extension groups (LAPCa, LPCa), risk groups, Gleason Score, ISUP, pT, or preoperative PSA value.

Insomuch as the TMPRSS2-ERG subtype results in a more homogeneous group, we performed DEA between the groups of LPCa (n = 16) and LAPCa (n = 18) samples only within the TMPRSS2-ERG subtype.

**Figure 3:**
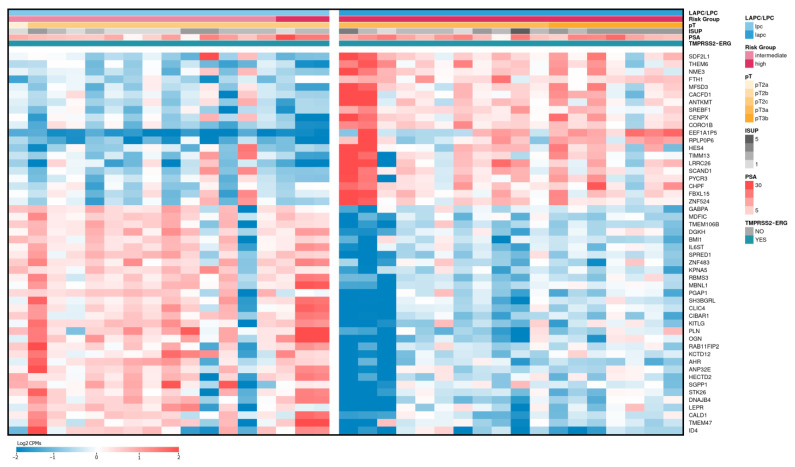
Heatmap of top 50 differentially expressed genes between LAPCa and LPCa for the TMPRSS2-ERG subtype. LAPCa—locally advanced PCa; LPCa—localized PCa. Red color indicates upregulated genes, blue—downregulated.

As a result, 207 DE genes were identified (*p*-values ≤ 0.05 according to the QLF and MW tests, Log2CPM ≥ 3, |Log2FC| ≥ 1, Supplementary Table S1-3). Figure 3 shows the expression profiles of the top 50 of these DE genes.

**Figure 4:**
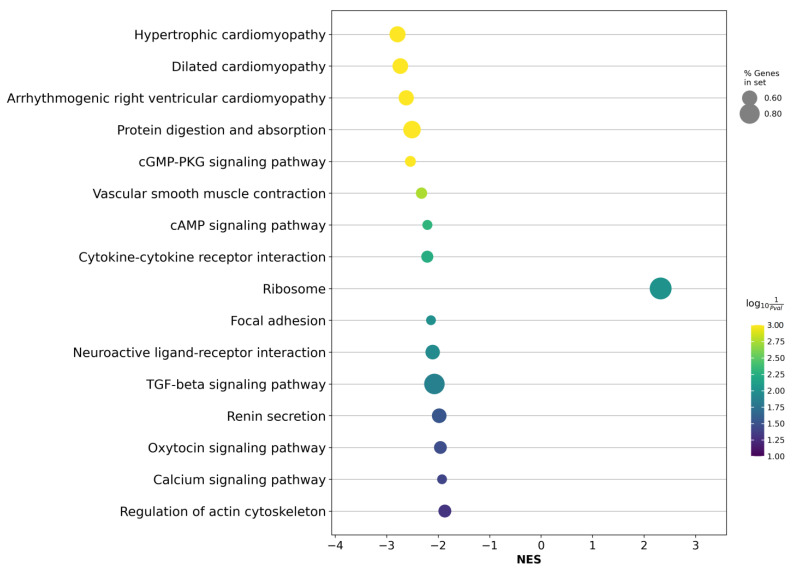
Dotplot of GSEA results illustrating KEGG-based biological processes associated with the LAPCa category versus LPCa for the high-risk group within the TMPRSS2-ERG molecular subtype. The figure shows statistically significantly enriched biological pathways based on the coexpression of genes. NES—normalized enrichment score.

Based on the identified DE genes, we also performed an enrichment analysis of a number of biological pathways. As a result of the analysis, we found statistically significant changes in 16 biological processes (FDR ≤ 0.05, Figure 4, Supplementary Table S2-2).

After clarifying the TMPRSS2-ERG molecular subtype in the LAPCa category, various signaling processes that have a known involvement in PCa progression, such as the “TGF-beta signaling pathway”, were seen to become the most significant. A complete list of analysis results is presented in Supplementary Table S2-2.

We also identified DE genes whose expression was statistically significantly associated with the LAPCa group within the TMPRSS2-ERG subtype: *BHLHA15*, *CIBAR1*, *CLIC4*, *CORO1B*, *CRB3*, *DNAJB4*, *DNM3OS*, *EEF1A1P5*, *HECTD2*, *ID4*, *MFSD3*, *MIR222HG*, *OGN*, *RPLP0P6*, *SH3BGRL*, and *ZNF483*. The results of the differential expression of these genes are presented in Table 1.

It should be noted that, according to the differential expression of the *CIBAR1*, *CLIC4*, *DNAJB4*, and *EEF1A1P5* genes within the TMPRSS2-ERG molecular subtype, we observed the highest correlation of these with the LAPCa group.

We also considered the predictive potential of selected genes by analyzing ROC curves based on a logistic regression algorithm. The results of the analysis are presented in Table 2.

According to the results obtained, the *EEF1A1P5* and *RPLP0P6* genes had the highest values (AUC > 0.9) of the AUC metric in the test data, both when analyzed in the total set of samples and within the TMPRSS2-ERG molecular subtype. It is also worth noting that an increase in value of more than 0.9 for the AUC metrics after clarification of the molecular subtype was found in the *CIBAR1*, *CLIC4*, *HECTD2*, *OGN*, and *ZNF483* genes.

### 2.4. Differentially Expressed Genes Associated with ISUP 3 at Intermediate Risk for Localized PCa

**Figure 5:**
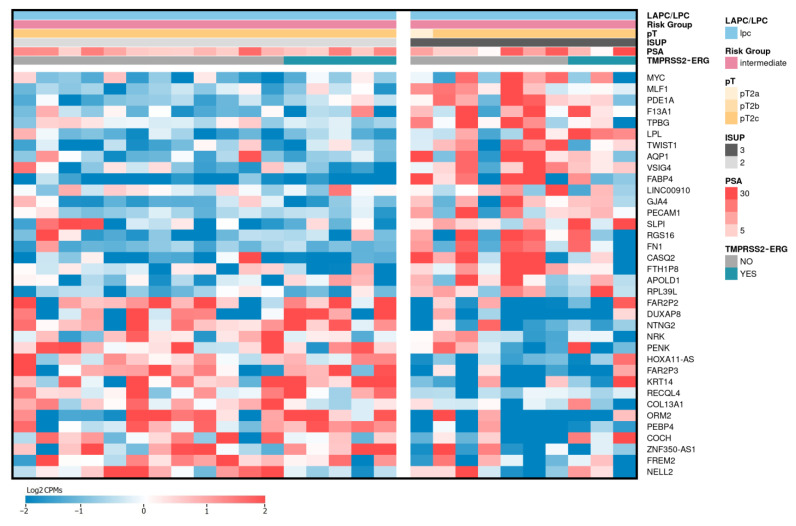
Heatmap of identified differentially expressed genes between ISUP 3 and ISUP 2 for LPCa. LPCa—localized PCa. Red color indicates upregulated genes, blue—downregulated.

Based on the RNA-Seq data obtained for ISUP = 3 (n = 17) and ISUP = 2 (n = 10) LPCa in our Russian patient cohort within the GS = 7, 36 differentially expressed (DE) genes were identified (*p*-values ≤ 0.05 according to the QLF and MW tests, Log2CPM ≥ 3, |Log2FC| ≥ 1, Supplementary Table S1-4). Figure 5 shows the expression profiles of these DE genes.

**Figure 6:**
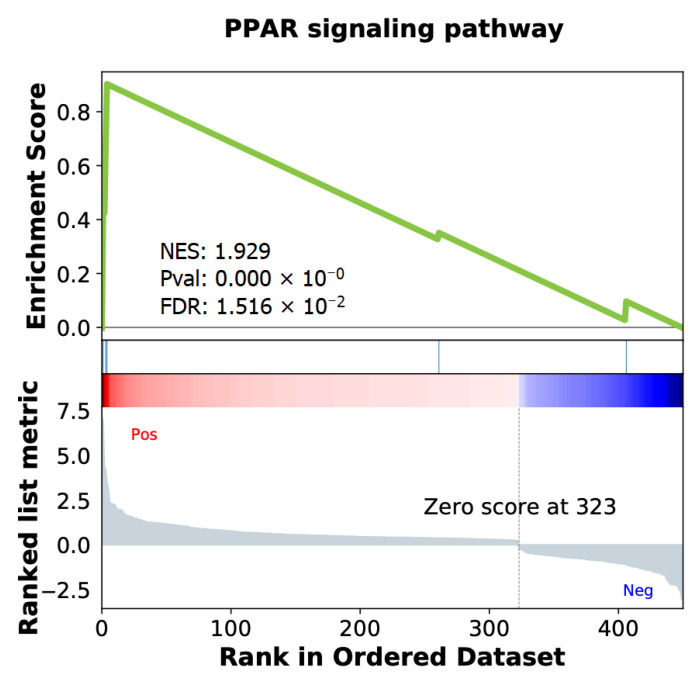
GSEA enrichment plot in ISUP 3 group versus ISUP 2 group in LPCa intermediate-risk group.

Based on the results of our analysis of the enrichment of biological pathways associated with the ISUP 3 group in the LPCa of the intermediate-risk group, we found statistically significant changes in only one cancer-associated biological process, the “PPAR signaling pathway” (NES = 1.93; FDR = 0.02, Figure 6). A complete list of analysis results is presented in Supplementary Table S2-3.

### 2.5. Validation of the Relative Expression of Genes Associated with the ISUP 3 Group in the Intermediate-Risk LPCa Group of Russian Patients

**Figure 7:**
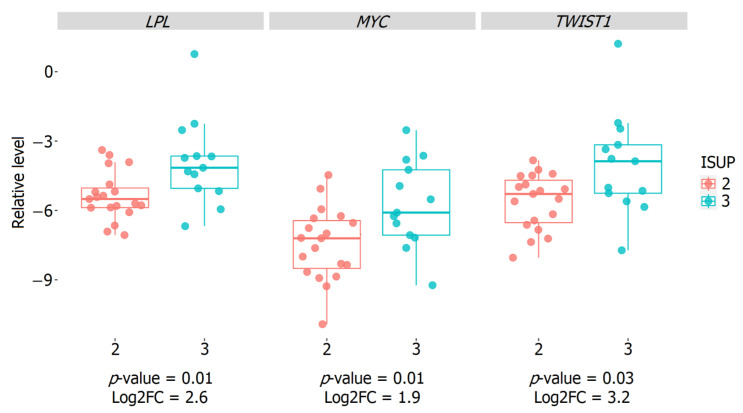
Boxplots of the relative expression of mRNA *LPL*, *MYC*, and *TWIST1* genes between the ISUP 3 and ISUP 2 groups in the LPCa intermediate-risk group of the Russian patient cohort. ISUP 2 group is marked in red, ISUP 3 group—blue.

We also carried out selection and validation of the relative expression of promising genes associated with the ISUP 3 group in our cohort of patients, in order that they might later be considered as additional prognostic markers. Based on the most significant differential expression results, the *LPL*, *MYC*, and *TWIST1* genes were selected for validation by qPCR. According to the results of this validation of the relative expressions of the genes under consideration, statistically significant results were confirmed (Figure 7).

## 3. Discussion

In the current work, we performed a comprehensive study of LPCa, considering the risk groups, the degree of differentiation of the tumor cells (ISUP classification), and inclusion in the TMPRSS2-ERG molecular subtype based on RNA-Seq profiling.

As our results demonstrate, only the PCa cases belonging to high- and intermediate-risk groups have insufficiently stable transcriptomic variations between themselves. We found changes in only six genes (↓*COL17A1*, ↑*FAM83D*, ↑*GCSAML*, ↓*IER2*, ↑*IFI44L*, ↓*MYH3*), but little is known about their involvement in PCa. However, when considering two grades of tumor extension (LPCa and LAPCa), we were able to find significant differences in gene expression. It is necessary to mention that when considering the TMPRSS2-ERG molecular subtype (i.e., when only TMPRSS2-ERG-positive cases are included in the analysis), the significance of the changes in the expression of the previously detected genes (*CIBAR1*, *CLIC4*, *EEF1A1P5*, *OGN*, *RPLP0P6*, and *ZNF483*) increased. 

The *CIBAR1*, *CLIC4*, *OGN*, and *ZNF483* are protein-coding genes, but nothing is known about their association with PCa and cancer in general. *EEF1A1P5* and *RPLP0P6* are pseudogenes, and there are currently no data on their association with PCa progression. However, there is evidence that *EEF1A1P5* gene transcripts and the RPLP0P6 protein are present in exosomes of various tumor cell lines [17,18]. 

Regarding the *HECTD2* gene in PCa, it has been shown that a decrease in the expression of this gene significantly affects androgen-induced and AR-mediated transcription, while suppression of *HECTD2* also enhances the growth of LNCaP cells [19]. According to our data, we also observed a decrease in the expression of the *HECTD2* gene in the more advanced stage of the high-risk group—LAPCa.

When considering the most significantly enhanced cancer-associated biological processes in the LAPCa category within the TMPRSS2-ERG molecular subtype, such signaling pathways as the “cAMP signaling pathway” and the “TGF-beta signaling pathway” come to the fore. Aberrant signaling in these pathways has been implicated in various types of tumor. Transforming growth factor *β* (TGF-*β*) is a key regulator of many biological processes, including metastasis and invasion. This protein binds to intracellular receptors and activates a signaling pathway that is involved in the regulation of cell proliferation and differentiation. Multiple studies have shown that such TGF-*β* signaling is associated with poor prognosis in PCa [20,21,22]. cAMP signaling can play both a tumor-suppressing and tumor-promoting role, depending on the type of tumor. This cascade can also be used to regulate the growth, migration, invasion, and metabolism of cancer cells, including those in PCa [23,24]. Thus, our results indicate that the identified signaling pathways play an important role in PCa and can potentially be used to assess likely prognoses. Further research is needed to better understand the mechanisms of action of these signaling pathways at different stages of PCa progression and their potential value in developing more effective treatments.

Our study also considered another clinical problem—that based on the molecular heterogeneity of prostate tumors. According to the D’Amico classification for LPCa, patients with PSA from 10 to 20 ng/mL, a Gleason score (GS) of 7 (ISUP 2/3), and cT2b belong to the intermediate-risk group. The standard of care for patients in this group is radical prostatectomy with/without extended pelvic lymphadenectomy or external beam radiation therapy.

This group of patients is one of the most heterogeneous, including both patients with GS (3+4) and GS (4+3), as well as a variety of PSA levels. The main difficulty in the treatment of patients in this risk group is the high probability of disease progression after radical treatment. The recommendations of the American Association of Urology (2017) propose a division of this intermediate-risk group into favorable (GS 3+4, risk group 2 (ISUP 2)) and unfavorable (GS 4+3, risk group 3 (ISUP 3)). Unfortunately, this division does not allow a qualitative change in the approach to the treatment of patients in this category, and the main approach remains radical prostatectomy or radiation therapy with androgen deprivation therapy.

We investigated the features of the transcriptome profile in our LPCa patients in the intermediate-risk group, based on the results of differential gene expression between the ISUP 2 and 3 groups.

From our analysis of biological pathway enrichment, it was found that the ISUP 3 group was characterized by a statistically significant increase in the regulation of the PPAR signaling pathway (NES = 1.93; FDR = 0.015). This signaling cascade is one of the most important mechanisms for regulating lipid and glucose metabolism, as well as cell growth and differentiation. The main participants in this pathway, according to our sample of patients, are the genes *ADIPOQ* (LogFC = 6.92; MW *p*-value = 0.04), *FABP4* (LogFC = 4.72; MW *p*-value = 0.03), and *LPL* (LogFC = 3.45; MW *p*-value = 0.01).

Based on the identified profiles of the DE genes, we selected the most significant of them in terms of statistical and expression metrics for subsequent qPCR validation: *LPL*, *MYC*, and *TWIST1*. Statistically significant differences between the ISUP 2 and ISUP 3 groups were shown, based on the relative expression of all the selected genes (*p*-value ≤ 0.05).

The *TWIST1* and *MYC* genes are well-known and important regulators of cancer-associated processes and remain objects of active research in the field of oncology. Various studies have shown a relationship between high levels of expression of these genes and the aggressiveness of oncological diseases, including PCa [25,26,27,28]. Based on our results with Russian patients, we have also demonstrated that increased expression of the *TWIST1* and *MYC* genes is associated with an unfavorable intermediate risk in LPCa.

However, the *LPL* gene, which encodes the enzyme lipoprotein lipase and plays a key role in the metabolism of fats and carbohydrates, is of particular interest [29]. *LPL* is an important enzyme that is secreted by extracellular lipolysis and can potentially be supplied by tumor cells or adjacent adipose tissue cells into the tumor microenvironment [30].

The main function of this enzyme is to hydrolyze triglycerides, resulting in the release of fatty acids, essential building blocks of biological membranes, so such dysregulation of fatty acid metabolism is a vital component of lipid metabolism reprogramming in cancer. Tumor cells can use circulating free fatty acids as an energy source through lipolysis, for membrane biosynthesis or in signaling processes [31].

There is also experimental evidence that lipogenesis in tumors, associated with increased expression of the fatty acid synthase gene (*FASN*), is strongly dependent on the activity and/or expression of important oncogenes and tumor suppressors, including *MYC*, which cooperates with the sterol regulatory element-binding proteins (SREBPs) and can induce in vitro and in vivo lipogenesis, thus playing an important role in initiating and maintaining oncogenic growth [32].

## 4. Materials and Methods

### 4.1. Materials

The PCa samples were obtained from Russian patients who had undergone surgical intervention in the P.A. Hertzen Moscow Oncology Research Center (a branch of the National Medical Research Radiological Center, Ministry of Health of the Russian Federation) between 2015 and 2020. All materials were collected and characterized by the organization’s Pathology Department according to the WHO Classification of Tumours of the Urinary System and Male Genital Organs [33]. Each sample contained a minimum of 70% of tumor cells. Following surgical resection, the tissue samples were immediately frozen and stored in liquid nitrogen. In the current study, we used 58 samples of LPCa (adenocarcinoma) samples obtained from patients who underwent surgical treatment but had not received neoadjuvant therapy (Table 3). Additionally, RNA-Seq data of 43 lymph node-negative LAPCa samples obtained in our previous study were included [34]. The samples have the following characteristics: no regional metastasis (N0 category); negative resection margins; any PSA value; and any Gleason score. Samples with the presence of regional metastasis (pN1) were not included in the study as they are characterized by a specific transcriptomic expression pattern.

### 4.2. Methods

#### 4.2.1. Total RNA Isolation, Library Preparation, and Next Generation Sequencing (NGS)

Samples of frozen tumor tissues were first homogenized using a MagNA Lyser device (Roche, Basel, Switzerland). Subsequent total RNA isolation was performed using the MagNA Pure Compact RNA Kit (Roche) on the MagNA Pure Compact System (Roche) according to the manufacturer’s protocol. The concentration of isolated total RNA was assessed on a Qubit 4.0 fluorimeter (Thermo Fisher Scientific, Waltham, MA, USA) using the Qubit RNA BR Assay Kit (Thermo Fisher Scientific). The RIN (RNA integrity number) parameter, which characterizes the integrity of RNA, was evaluated using an Agilent 2100 Bioanalyzer (Agilent Technologies, Santa Clara, CA, USA). The RIN for all samples studied was no less than 7.

Sample preparation of mRNA libraries was performed using the TruSeq Stranded mRNA Kit (Illumina, San Diego, CA, USA) as described previously [35]. The size of the resulting mRNA library was ~260 bp.

High-throughput sequencing of mRNA libraries was performed on a NextSeq 500 System (Illumina) using NextSeq 500/550 High Output Kit v2.5 (Illumina) in 75 bp single-ended read mode. On average, about 20 million reads were received for each sample.

#### 4.2.2. Bioinformatic Analysis

FastQC (v.0.11.9, Cambridge, UK) (Babraham Bioinformatics-FastQC A Quality Control Tool for High throughput Sequence Data, accessed on 3 March 2021; available online, https://www.bioinformatics.babraham.ac.uk/projects/fastqc/) and Trimmomatic (v.0.33, Jülich, Germany) [36] were used for the quality control and trimming of reads, respectively. The STAR splice-aware aligner (v.2.7.1, Cold Spring Harbor, NY, USA) [37] was used to map the reads to the reference genome (GRCh37.p13, GENCODE, Cambridge, UK) (GENCODE—Human Release 19, accessed on 25 April 2021; available online, https://www.gencodegenes.org/human/release_19.html). FeatureCounts (Subread package v.1.6.4, Parkville, VIC, Australia) [38] was used to calculate the read counts per gene.

Differential expression analysis was carried out in the R environment (v.3.6.3, Vienna, Austria) (R: The R Project for Statistical Computing, accessed on 3 March 2023; available online, https://www.r-project.org/) using the edgeR package (v.3.24.3, NSW, Australia) [39], as described previously [34]. The results were considered significant at *p*-values of the quasi-likelihood F-test (QLF) and the Mann-Whitney U-test (MW) ≤ 0.05.

Gene set enrichment analysis (GSEA) was performed using the GSEApy package in Jupyter Notebook, Python (v.3.6) [40]. Annotation of the results was obtained, based on the KEGG Human 2021. The results were considered significant at FDR ≤ 0.05.

ROC analysis was performed on the basis of the Logistic Regression algorithm, using the scikit-learn library in Jupyter Notebook, Python (v.3.6).

#### 4.2.3. Reverse Transcription and Quantitative PCR (qPCR)

cDNA samples were obtained from the mRNA template using Mint reverse transcriptase and oligo(dT) primer (20 µM) according to the manufacturer’s protocol (Evrogen, Moscow, Russia). 

qPCR was performed in three technical replicates in total reaction volume of 10 μL on an Applied Biosystems 7500 instrument (Thermo Fisher Scientific). The TaqMan Gene Expression Assay Hs03063375_ft (Thermo Fisher Scientific) was used to determine the presence of the TMPRSS2-ERG fusion transcript. ROX was used as a reference dye. The *PUM1* gene was used as a reference for analysis of relative mRNA expression. The sequences of primers used to validate markers based on mRNA expression are shown in Table 4.

The following process was used for amplification: 95 °C for 15 min; 40 cycles at 95 °C for 15 s; and 60 °C for 60 s. To assess the level of expression, the method of relative measurements (*Δ*CT) was used and calculations were performed using the ATG program (Analysis of Transcription of Genes) [41]. Visualization and statistical analysis of expression results were performed using the MW test in the R environment (v.3.6.3, Vienna, Austria).

## 5. Conclusions

We performed RNA-Seq profiling of 58 LPCa and 43 LAPCa tissue samples, obtained as a result of radical prostatectomy on a cohort of Russian patients. Bioinformatics analysis revealed that in the high-risk group, LAPCs showed enrichment of certain biological pathways, both across the entire sample and within the TMPRSS2-ERG molecular subtype. Such enrichment could be further investigated in the search for new potential therapeutic targets in the studied categories of PCa. We also determined which genes showed the greatest significant differences in expression, allowing the LPCa and LAPCa categories in the high-risk group to be distinguished, when taking into account the TMPRSS2-ERG molecular subtype. The highest predictive potential was found for the *CIBAR1*, *CLIC4*, *EEF1A1P5*, *OGN*, *RPLP0P6*, and *ZNF483* genes.

The study also examined the transcriptomic features of the intermediate-risk group of LPCa, within GS = 7 (ISUP classification—groups 2 and 3). A statistically significant enrichment of the “PPAR signaling pathway” in the ISUP 3 group was shown. Based on the identified transcriptomic profile, the *LPL*, *MYC*, and *TWIST1* genes were selected as promising additional prognostic markers, the statistical significance of which was confirmed based on qPCR validation.

## Figures and Tables

**Table 1:** Differential expression of genes statistically significantly associated with the LAPCa group, including within the TMPRSS2-ERG subtype. LAPCa—locally advanced prostate cancer, LPCa—localized prostate cancer, Log2FC—log2 transformed fold change, *r_s_*—Spearman correlation coefficient, grey background—downregulated genes.

Gene Name (Biotype)	Description	LAPCa vs. LPCa		LAPCa vs. LPCa (TMPRSS2-ERG)	
		Log2FC	*r_s_*	Log2FC	*r_s_*
*BHLHA15* (protein coding)	basic helix-loop-helix family member a15	1.79	0.57	1.40	0.68
*CIBAR1* (protein coding)	CBY1 interacting BAR domain containing 1	−1.05	−0.65	−1.20	−0.81
*CLIC4* (protein coding)	chloride intracellular channel 4	−1.02	−0.53	−1.14	−0.81
*CORO1B* (protein coding)	coronin 1B	1.17	0.63	1.04	0.77
*CRB3* (protein coding)	crumbs cell polarity complex component 3	1.15	0.58	1.03	0.72
*DNAJB4* (protein coding)	DnaJ heat shock protein family (Hsp40) member B4	−1.13	−0.62	−1.22	−0.80
*DNM3OS* (lncRNA)	DNM3 opposite strand/antisense RNA	−1.22	−0.62	−1.58	−0.75
*EEF1A1P5* (processed pseudogene)	eukaryotic translation elongation factor 1 alpha 1 pseudogene 5	2.45	0.77	2.70	0.87
*HECTD2* (protein coding)	HECT domain E3 ubiquitin protein ligase 2	−1.05	−0.63	−1.04	−0.77
*ID4* (protein coding)	inhibitor of DNA binding 4, HLH protein	−1.34	−0.55	5.85	−0.67
*MFSD3* (protein coding)	major facilitator superfamily domain containing 3	1.10	0.61	1.09	0.73
*MIR222HG* (lncRNA)	miR222/221 cluster host gene	−1.27	−0.56	−1.27	−0.60
*OGN* (protein coding)	osteoglycin	−1.10	−0.54	−1.62	−0.76
*RPLP0P6* (processed pseudogene)	ribosomal protein lateral stalk subunit P0 pseudogene 6	1.83	0.70	1.94	0.77
*SH3BGRL* (protein coding)	SH3 domain binding glutamate rich protein like	−1.06	−0.63	−1.12	−0.76
*ZNF483* (protein coding)	zinc finger protein 483	−1.02	−0.58	−1.15	−0.78

**Table 2:** Results of ROC analysis based on the logistic regression algorithm for test data based on a cohort of Russian patients with high-risk LPCa and LAPCa, considering the TMPRSS2-ERG molecular subtype. LAPCa—locally advanced prostate cancer, LPCa—localized prostate cancer, AUC—Area Under Curve, CI—confidence interval, grey background—downregulated genes.

Gene Name	LAPCa vs. LPCa			LAPCa vs. LPCa (TMPRSS2-ERG)		
	AUC (CI, 95%)	Accuracy	*p*-Value	AUC (CI, 95%)	Accuracy	*p*-Value
*BHLHA15*	0.864 (0.581–0.964)	0.750	6.0 × 10^−6^	0.867 (0.820–1.000)	0.708	1.2 × 10^−2^
*CIBAR1*	0.821 (0.701–1.000)	0.708	3.0 × 10^−6^	0.979 (0.877–1.000)	0.917	8.8 × 10^−3^
*CLIC4*	0.711 (0.611–0.985)	0.750	8.1 × 10^−5^	0.930 (0.891–1.000)	0.833	8.5 × 10^−3^
*CORO1B*	0.830 (0.701–1.000)	0.750	1.0 × 10^−6^	0.889 (0.897–1.000)	0.792	2.6 × 10^−2^
*CRB3*	0.798 (0.661–1.000)	0.750	2.0 × 10^−6^	0.734 (0.686–1.000)	0.542	6.0 × 10^−3^
*DNAJB4*	0.867 (0.654–0.992)	0.792	1.0 × 10^−5^	0.824 (0.790–1.000)	0.708	8.4 × 10^−3^
*DNM3OS*	0.867 (0.718–1.000)	0.792	1.2 × 10^−5^	0.824 (0.807–1.000)	0.708	9.7 × 10^−3^
*EEF1A1P5*	0.979 (0.904–1.000)	0.917	6.5 × 10^−5^	1.000 (0.977–1.000)	0.875	2.9 × 10^−2^
*HECTD2*	0.815 (0.691–1.000)	0.708	6.0 × 10^−6^	0.977 (0.947–1.000)	0.875	5.7 × 10^−3^
*ID4*	0.832 (0.486–0.924)	0.750	3.9 × 10^−5^	0.811 (0.592–1.000)	0.750	8.3 × 10^−3^
*MFSD3*	0.837 (0.604–0.992)	0.667	2.0 × 10^−5^	0.874 (0.754–1.000)	0.708	8.5 × 10^−3^
*MIR222HG*	0.778 (0.512–0.950)	0.708	1.3 × 10^−4^	0.879 (0.763–1.000)	0.750	4.1 × 10^−2^
*OGN*	0.764 (0.541–0.988)	0.708	3.9 × 10^−5^	0.963 (0.797–1.000)	0.875	1.6 × 10^−2^
*RPLP0P6*	0.930 (0.745–1.000)	0.792	5.2 × 10^−6^	0.984 (0.764–1.000)	0.958	2.3 × 10^−2^
*SH3BGRL*	0.857 (0.581–0.981)	0.792	6.0 × 10^−6^	0.818 (0.757–1.000)	0.792	9.2 × 10^−3^
*ZNF483*	0.879 (0.501–0.944)	0.750	1.5 × 10^−5^	0.914 (0.891–1.000)	0.708	7.3 × 10^−3^

**Table 3:** Clinical and pathological characteristics of the studied cohort. pT—primary tumor estimation; N—regional lymph nodes; M—distant metastases; ISUP—The International Society of Urological Pathology.

Criterion		LPCa, n	LAPCa, n
PCa samples		58	43
Age, years		64 (41–78)	64 (46–77)
pT	pT2a	3	0
	pT2b	5	0
	pT2c	50	0
	pT3a	0	30
	pT3b	0	13
	pT4	0	0
pN	pN0	58	43
	pN1	0	0
cM	cM0	58	43
	cM1	0	0
Gleason score	6	22	6
	7	32	29
	8	2	5
	9	1	3
	10	0	0
ISUP grade	1	22	6
	2	20	17
	3	12	12
	4	2	5
	5	1	3
PSA, ng/mL		11.1 (0.3–30)	12.8 (3.4–27.6)
Risk group	Low	1	0
	Intermediate	47	0
	High	10	43
Molecular subtype TMPRSS2-ERG	Yes	16	18
	No	42	25

**Table 4:** Primer sequences for assessing the level of mRNA expression.

mRNA	Primer Sequence (5′→3′)	Product Length, b.p.
*LPL*	F: CAGCCCTACCCTTGTTAGTTATT R: ACGTTGGAGGATGTGCTATTT	103
*MYC*	F: ATCTCTGGGAGGAATGCTACTA R: ATCTGCGTGGCTACAGATAAG	95
*TWIST*	F: CGGAGACCTAGATGTCATTGTTT R: ACGCCCTGTTTCTTTGAATTTG	146
*PUM1*	F: TGGACCATTTCGCCCTTTAG R: CAGAGAGTTGTTGCCGTAGAA	103

## Data Availability

All data generated or analyzed during this study are available (GSE229904). The dataset also includes expression data from our previous studies devoted to the analysis of primary PCa tumors.

## Supplementary Materials

The supporting information can be downloaded at https://www.mdpi.com/article/10.3390/ijms24119282/s1.
